# Strategy Diversity Stabilizes Mutualism through Investment Cycles, Phase Polymorphism, and Spatial Bubbles

**DOI:** 10.1371/journal.pcbi.1002660

**Published:** 2012-11-15

**Authors:** Gergely Boza, Ádám Kun, István Scheuring, Ulf Dieckmann

**Affiliations:** 1 Evolution and Ecology Program, International Institute for Applied Systems Analysis (IIASA), Laxenburg, Austria; 2 MTA-ELTE-MTM Ecology Research Group, Eötvös Loránd University, Budapest, Hungary; 3 Parmenides Center for the Study of Thinking, Munich/Pullach, Germany; 4 Department of Plant Systematics, Ecology, and Theoretical Biology, Institute of Biology, Eötvös Loránd University, Budapest, Hungary; 5 MTA-ELTE Research Group in Theoretical Biology and Evolutionary Ecology, Eötvös Loránd University, Budapest, Hungary; University of Washington, United States of America

## Abstract

There is continuing interest in understanding factors that facilitate the evolution and stability of cooperation within and between species. Such interactions will often involve plasticity in investment behavior, in response to the interacting partner's investments. Our aim here is to investigate the evolution and stability of reciprocal investment behavior in interspecific interactions, a key phenomenon strongly supported by experimental observations. In particular, we present a comprehensive analysis of a continuous reciprocal investment game between mutualists, both in well-mixed and spatially structured populations, and we demonstrate a series of novel mechanisms for maintaining interspecific mutualism. We demonstrate that mutualistic partners invariably follow investment cycles, during which mutualism first increases, before both partners eventually reduce their investments to zero, so that these cycles always conclude with full defection. We show that the key mechanism for stabilizing mutualism is phase polymorphism along the investment cycle. Although mutualistic partners perpetually change their strategies, the community-level distribution of investment levels becomes stationary. In spatially structured populations, the maintenance of polymorphism is further facilitated by dynamic mosaic structures, in which mutualistic partners form expanding and collapsing spatial bubbles or clusters. Additionally, we reveal strategy-diversity thresholds, both for well-mixed and spatially structured mutualistic communities, and discuss factors for meeting these thresholds, and thus maintaining mutualism. Our results demonstrate that interspecific mutualism, when considered as plastic investment behavior, can be unstable, and, in agreement with empirical observations, may involve a polymorphism of investment levels, varying both in space and in time. Identifying the mechanisms maintaining such polymorphism, and hence mutualism in natural communities, provides a significant step towards understanding the coevolution and population dynamics of mutualistic interactions.

## Author Summary

Mutualistic interactions between species are often best understood as gradually adjustable reciprocal investments made continuously or iteratively between participants. Prime examples are the mycorrhizal and rhizobial mutualisms so strongly affecting the productivity of plants. When such interactions are described by continuous reciprocal investment games, participants adjust their investments plastically in response to their mutualistic partner's most recent investment. Although common sense suggests that such conditional or reactive behavior provides a potent defense against exploitation, our comprehensive model analysis reveals that the coevolution of investment strategies will often instead induce instability and decay of mutualistic interactions. We also identify several factors that can prevent this decay. First, mutualisms can be stably maintained if the investment strategies of participants are sufficiently diverse. Second, if participants are limited in their movements, the formation of dynamic spatial mosaic structures promotes strategy diversity and thereby facilitates the maintenance of mutualism. These ecological and evolutionary dynamics result in communities with a diversity of interaction types, ranging from mutually beneficial to exploitative, and varying both in space and in time.

## Introduction

Investigating factors that promote cooperation is one of the main topics in evolutionary biology [1], [2]. Cooperation, a costly act that provides benefit for a partner [3], [4], is widespread in nature [5]–[7] and has been essential in shaping our biosphere [8], [9].

The basic dilemma of intraspecific cooperation [10] also applies to interspecific mutualism [2], [5]: while both partners of such interactions would be better off helping each other, a cheater that accepts help without reciprocating will have higher fitness and thus spread in the population [11]–[14]. Cheating consistently committed by one partner can shift a mutualistic interaction into parasitism [15], as corroborated by observations in ant–plant mutualisms [16]–[18] or mycorrhizal mutualisms [19], [20].

Despite the underlying dilemma being similar, interspecific cooperation differs from intraspecific cooperation in several key features. In interspecific cooperation, the interaction is under the control of two separate genomes, the evolutionary success of strategies in one species directly depends on the strategies in its partner species [21], [22] rather than on those on its own species, and the spread of a successful strategy in one species does not automatically result in the spread of a matching strategy in the other. Another consequence of partners belonging to different species is that one important mechanism promoting cooperation, kin selection [1], [2], cannot play a role. Furthermore, in many mutualisms, the partners occupy different niches [5], and are thus not in direct competition with each other. For all these reasons, models of intraspecific cooperation do not cover the specificities of mutualisms, so that mechanisms promoting mutualism have to be explored and identified separately [14], [22].

Knowing the costs and benefits of a mutualistic interaction is fundamental for understanding its ecology and evolution [13], [23]. Commonly studied examples are nutritional mutualisms, such as mycorrhiza [24]–[26] or rhizobia [27], and other forms of symbiosis, including endosymbiosis [9]. However, these interactions are often not described by a single discrete event, but involve the long-term, often continuous, exchange of goods (such as in rhizobia–plant interactions) [28], [29]. Quantifying the effective costs and benefits of these recurrent, and often reactive or conditional, exchanges is more complicated. For example, experiments found that the volume of nitrogen-containing substances provided by the nitrogen-fixing bacteria (such as ammonium, aspartate, or alanine) is increased by the concentration of oxygen and carbohydrates (such as succinate or glutamate) provided and controlled by the host plant [30], [31]. In turn, from the perspective of the plant, higher nitrogen supply via fixation can enhance plant metabolism [27], [32], which can translate into higher carbohydrate supply to the symbiont [28]. Many studies have revealed similar mechanisms for the conditional exchange of nutrients (such as phosphates and carbohydrates) in mycorrhizal symbiosis [19], [33]–[35]. Such long-term (even lifelong) associations allow partners continuously to adjust their investments into the mutualistic interaction [36]. Individuals may increase or decrease rewards in response to increased or decreased services received from a partner [18], [28], [34], [37]–[39]. This iterative reciprocation throughout an interaction obviously involves phenotypic plasticity of the traits involved in the interaction [40], [41] and offers a control mechanism between the partners [42]. Akin to reaction norms, which describe how the environment can affect a genotype's expression [41], the rule of reciprocation can be described by an interaction norm [40], which thus characterizes the expression of a trait as a function of the interacting partner's strategy.

In spite of the biological importance of, and the wealth of information available for, interspecific cooperation, the evolutionary dynamics of mutualism are far less understood [13], [14]. Moreover, among models of mutualism, few concentrate on the evolutionary dynamics of interactions on the individual level when there is continuous feedback between the partners [42], as captured by the concept of partner fidelity feedback [1], [11]. One of the few existing models addressing this challenge is the one proposed by Doebeli and Knowlton [43], which is among the three most cited evolutionary models in the mutualism literature (along with biological market models [44]; and models of geographic mosaic theory of coevolution [45]). In their individual-based model, each individual's strategy is characterized by two values: the so-called initial offer and the reward rate. The initial offer amounts to an unconditional or fixed investment in the mutualistic interaction, whereas the reward rate quantifies a conditional or variable component, which determines how an individual's investment depends on the payoff it gained from its current partner in the previous round. This distinction is well founded in the biology of mutualistic interactions. For example, in mutualistic interactions involving ants defending their mutualistic partners from predation, as in the case of ants and lycaenid butterfly larvae [46] or aphids [14], both partners can adjust their investments by providing less nectar or less tending. There is also an unconditional initial investment in many interactions, which is required for establishing an interaction with a partner before evaluating its quality as a mutualist [14]. Examples include honeydew droplets or volatile substances from tentacle organs to attract partners [17] or chemical compounds released by plants in mycorrhizal or rhizobial mutualisms [27]. Moreover, creating an interface for physical contact sometimes requires high investments from both parties before an exchange of nutrients can commence [47].

Doebeli and Knowlton [43] concluded that population structure or spatial confinement is essential for stabilizing mutualisms. They elegantly demonstrated that without the facilitating effect of space, mutualistic investments vanish from the populations. Nevertheless, the specific role of spatial structure and the differences in the dynamics of mutualism in spatially structured and well-mixed populations need to be still more deeply understood. Moreover, Doebeli and Knowlton's conclusion regarding the necessity of spatial population structure was based on a single example. Reviews of the mutualism literature [13], [14], [38] have therefore debated the importance of space in stabilizing mutualism, and independent theoretical studies [37] could not corroborate the necessity of space for stabilizing mutualism. What are the causes for this apparent discrepancy? Are mutualisms really unstable in the absence of spatial structure? Our aim here is to unravel the role of space in the evolutionary dynamics of mutualism and to provide a platform for connecting model results with experimental findings.

## Methods

Throughout this study, we closely follow the seminal model introduced by Doebeli and Knowlton [43]. We define mutualism as an interaction between individuals from different species, Mutualist A and Mutualist B. We highlight that Mutualist A and Mutualist B in our model can, more generally, be interpreted as mutualist guilds: such guilds are composed of one species or several species that share the same functional relationship with the other guild. The fitness of an individual depends on the outcome of its interaction with a member of the other mutualist guild, while competition occurs only between members of the same guild. Modeling the latter as competition for space, the populations of the two mutualist guilds can be conceived as occupying two separate square lattices. We do not consider sexual reproduction, and the only characteristics of individuals we examine are the traits affecting their mutualistic investments, as detailed below.

### Mutualistic investments

Each individual's strategy for interacting with individuals from the other guild is specified by two (non-negative) quantitative adaptive traits: an unconditional investment 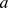, determining the initial offer to be made to a partner, and a conditional investment 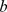, determining the reward rate according to which investment received from a partner are reciprocated. Thus, the strategy of Mutualist A is given by the pair (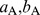), and the strategy of Mutualist B is given by the pair (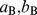). The initial offer is an unconditional and fixed investment into the mutualistic interaction, whereas the reward rate determines how the investment changes depending on the last payoff gained from the interaction with the current partner.

### Interactions and payoffs

Payoffs are calculated through an iterative procedure, based on a fixed number 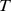 of iterations, or interaction rounds. Following Doebeli and Knowlton [43], we use 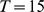 rounds. Before the first iteration, the payoffs of all individuals are set to zero. Below we consider the investments made, costs incurred, benefits received, and payoffs accrued by a mutualist with strategy 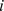 interacting with a mutualist with strategy 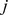. In the first iteration 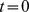, the investment 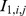 is simply given by the trait 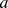, 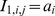. In every subsequent iteration 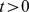, the investment 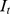 is determined by a linear reactive strategy,
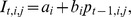
where 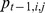 is the net benefit, or payoff, obtained in the previous iteration 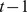 by strategy 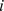 interacting with strategy 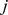 (see below for further details on how partners are chosen). Investments 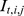 are always non-negative: if they would be negative, they are set to zero.

The payoffs are calculated from the investments made by the individuals of Mutualist A and Mutualist B. Each investment implies a cost for the donor and a benefit for the receiver,
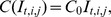


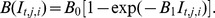
Accordingly, the payoff from one iteration of the interaction is
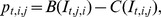
where 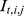 and 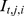, respectively, are the investments of the focal individual 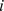 and of its partner 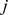 in round 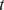 [43]. Total payoffs are obtained by summing payoffs over all rounds of the mutualistic interaction, 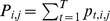.

### Benefit-to-cost relationship

Compared to traditional game theoretical models, for which the benefit-to-cost ratio is given by 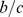 (benefit divided by the cost of cooperation), for the current model, it is much harder to define the benefit-to-cost relationship, because of the nonlinear benefit function and the complex iterated nature of the game. It is therefore helpful to examine an approximation for infinitesimally small investments: in this case, the benefit function simplifies to 
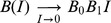
. We can then consider the benefit-to-cost ratio 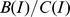 in this limit, which gives 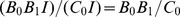. This simple expression serves as an upper bound: for higher investments, the nonlinearity of benefits causes the benefit-to-cost ratio always to fall below 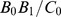. Hence, for our model, a higher benefit-to-cost ratio means a higher product of the two parameter values for the benefit function compared to the parameter value for the cost function.

### Competition

In the spatial model, the focal individual and its 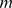 closest neighbors (we use the Moore neighborhood with 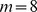) compete for the focal site. In the well-mixed model, we randomly draw as competitors 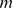 individuals from the focal individual's whole population. We employ either of two update rules. With “best takes over” updating, which was also used by Doebeli and Knowlton [43], the individual with the highest payoff replaces the focal individual [43], [48], [49]. This implies that, if no competitor has a higher payoff than the focal individual, the later stays unchanged. If two individuals have the same payoffs, the winner is randomly chosen between them. With “pairwise comparison” updating, a random competitor 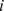 (interacting with individual 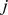) replaces the focal individual 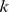 (interacting with individual 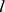) with probability 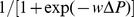, depending on their payoff difference 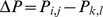 [50], [51]; for scaling the strength of selection, we use 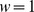. Both rules belong to the class of so-called death–birth updating processes [51].

### Mutation

The two traits can mutate independently with probability 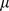 per update. The mutant trait value is drawn from a normal distribution, with a mean equaling the current trait value and a given variance. Doebeli and Knowlton [43] assumed that the standard deviation 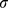 of this normal distribution is a given percentage (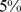) of the current trait value. This assumption implies that the coefficient of variation (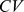) is constant; thus, for smaller trait values the resultant variance is smaller than for larger trait values. Accordingly, when a trait value approaches 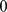, its mutational variance also approaches 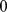. This means that trait values can essentially get “stuck” close to 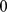. To evaluate the consequences of this effect, we also consider models in which the mutational standard deviation is kept fixed (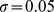).

### Updating

In our model, 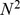 updates occur per generation, where 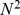 is the population of Mutualist A and Mutualist B. In the spatial model, 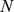 is the width and height of the square lattice (we consider values 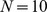, 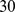, 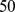, and 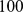). For each update, we choose an interacting pair of Mutualist A and B. In the spatial model, the chosen individuals that occupy matching sites on the two lattices, whereas in the well-mixed model, they are randomly drawn from the two lattices. With synchronous updating, all individuals are updated at once, while with asynchronous updating, randomly chosen individuals are updated. Unless mentioned otherwise, we use asynchronous updating. Each update starts with an update of the payoffs of the involved individuals, followed by competition among them.

We initialize the model dynamics with two homogeneous populations with both trait values close to 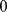 (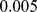, unless indicated otherwise), implying that individuals are not mutualistic. We also consider different initial conditions, with one or both of the traits set to higher values (chosen from the interval 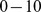). We then run the dynamics for 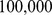 generations (unless otherwise indicated), which is a time horizon chosen to be long enough to detect the main dynamical trends for all considered model settings.

## Results

As the dynamics of the full model are highly complex, we gradually build up understanding by analyzing model versions of increasing complexity throughout the next five sections, starting with the simplest model version that still retains key dynamical features of the full model, and after five steps eventually arriving back at the full model. Based on the payoffs defined above, we start from a best-response analysis of the mutualistic investment strategies; we then examine the selection pressures on these investments for mutualists with low polymorphism, consider the individual-based model without spatial population structure but with higher degrees of polymorphism, reinstate the spatial population structure, and finally conclude our analysis of the full model by exploring the effects of different mutation schemes and update rules. The insights gained through this five-step investigation allow us to revisit and reinterpret the results by Doebeli and Knowlton [43] at the conclusion of this section.

### No investment as a best-response equilibrium

As a first step, we determine best-response equilibria of the mutualistic investments. The interspecific best response 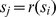 is defined here as the strategy 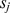 of mutualist 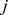 that has the highest payoff playing against strategy 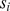 in the other mutualist guild, for 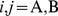, 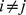. Thus, investment strategies are in a best-response equilibrium 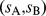, if 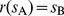 and 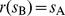, if, that is, these strategies are the best responses to each other. Incidentally, this implies 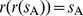, which highlights a similarity with the concept of Nash equilibrium in intraspecific games; in that case, a strategy simply is the best response to itself [52].

As an analytical derivation of the best-response function 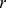 is not possible for our model, we calculate it numerically by fixing a strategy 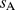 for Mutualist A, and then scan the two-dimensional strategy space of Mutualist B for the strategy 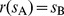 that yields the highest payoff to Mutualist B. We find that the best response to no investment is no investment, 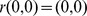, which therefore is a best-response equilibrium. The intuitive explanation is simple: when a partner does not reciprocate, the best strategy is not to invest in that partner. Furthermore, as our numerical investigations reveal, 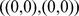, i.e., no investment by both mutualists (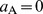, 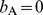, 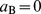, and 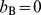), is the only best-response equilibrium of our model.

**Figure 1:**
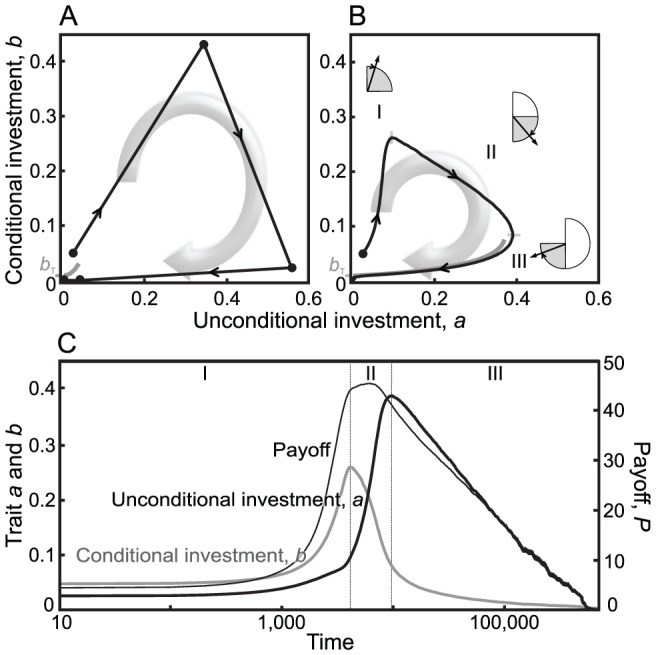
Illustration of investment cycle and reciprocation threshold in well-mixed communities. (A) Best-response dynamics. Arrows indicate the succession of best responses, leading to 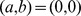 in just four steps. (B) Evolutionary dynamics in a community with low degrees of polymorphism and “pairwise comparison” updating. Arrows indicate changes of the selection gradient along the investment cycle. In A and B, representative evolutionary trajectories are shown starting above the reciprocation threshold (thick gray lines). (C) Resultant changes of investment traits and payoffs along the investment cycle in B. Results in B and C are averaged over 15 replicate model runs for the same initial condition. Parameters: 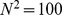, 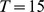, 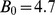, 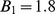, 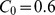, 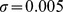, and 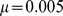.

Analyzing the local stability around this equilibrium, we find two types of local best-response dynamics. The equilibrium 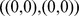 is locally stable [53], but strategies converge there only if they start out below a threshold level 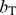 of reciprocation (gray lines in Figure 1*A and B*). Using the same approximation as Killingback and Doebeli [54] for small investments, we find that this threshold is determined by the slopes of the benefit and cost functions at zero investment, 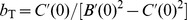 [53], [54], with 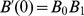 and 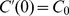 for our model. Thus, when starting out below 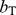, best-response strategies converge to the no-investment equilibrium, whereas when strategies start out above 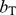, best responses lead to an increase in investment levels.

To understand the latter behavior, we consider the global best-response dynamics, which gives us full information about the coevolutionary changes we must expect in mutualistic investment strategies. For this, we start from the initial strategy of one mutualist, determine the optimal strategy of its partner, then again determine the optimal strategy of the first mutualist, and so on (Figure 1*A*). Interestingly, this shows that the no-investment strategy is not always the best response: above the thick gray line in Figure 1*A*, the best response differs from 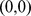 and causes reciprocation to increase in the first step (Figure 1*A*). After a few best-response steps, however, the dynamics always converge to the no-investment equilibrium, which is thus a global attractor of the best-response dynamics.

In conclusion, when the best-response dynamics start out below the threshold line, these dynamics will directly lead to the no-investment equilibrium, whereas when the initial strategies lie above the threshold line, the best-response dynamics will cause investments to increase temporarily, before bringing them down to 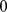 eventually (Figure 1*A*). Throughout this study, we refer to the latter behavior as the investment cycle.

### Investment cycle and selection pressures on mutualistic investments

We now show how our insights from the best-response analysis above extend to individual-based evolutionary dynamics under low degrees of polymorphism (Figure 1*B, C*). We find that when started below a threshold line (thick gray line in Figure 1*B*), the evolutionary dynamics monotonically converge to no investment. Above that line, the evolutionary dynamics temporarily drive investments up (Figure 1*B* and *C*). After these investments have passed a maximum, they monotonically converge to zero. In other words, we again find a “boom and bust” kind of investment cycle.

We can obtain the threshold of increasing investments (thick gray line in Figure 1*B*) in the limit of vanishing polymorphism. In that case, the selection pressures on the investment traits are given by 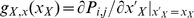, where 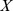 is the focal mutualist (A or B), 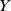 is the other mutualist (B or A, respectively), 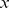 is the focal trait (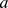 or 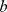), 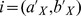 is the strategy of a mutant in 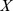, (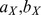) is the resident strategy in 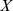, and 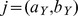 is the resident strategy in 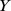. Positive selection pressures mean that mutants with increased trait values have higher payoffs than the current resident, and therefore can spread in the population. This kind of evolutionary dynamics is still simplified compared with an individual-based model; it yields good approximations only when population dynamics are sufficiently faster than trait dynamics (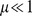), so mutants mostly encounter monomorphic populations, and when mutational steps are sufficiently small (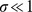), so the derivatives defining the selection pressures carry sufficient information for predicting the fate of all arising mutants. The obtained threshold line (thick gray line in Figure 1*B*) is the unstable part of the evolutionary isocline for trait 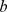, along which the selection pressure on 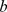 passes 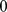 and thus changes sign. For small investments, and thus for 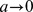, this isocline is located at 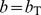.

We find that our aforementioned results regarding the investment cycle are robust. First, we can approximate the underlying individual-based evolutionary dynamics by adaptive dynamics theory [55], using the selection pressures 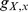 defined above. For low mutation probabilities 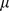 and standard deviations 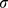, this approximation is accurate. Second, we can consider “best takes over” updating in an individual-based model with low degrees of polymorphism, and third, we can use a modification of this updating, so that the most successful mutant is drawn from a circle around the resident traits (for this, we sample random combinations of mutants from a circle of radius 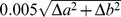, where 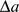 and 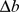 denote the trait differences between mutants and residents, and choose the one mutant with the highest payoff). All three of these variants yield results in agreement with those summarized above.

The emergence of the investment cycle can best be understood by examining the gradual coevolution of the two investment traits. Evolution starts from a slightly reactive state (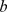 exceeds the threshold 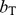), and both the unconditional and conditional investments first increase, as selection pressures are positive on both traits. Higher reactivity (resulting from higher conditional investment 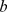) selects for a higher initial investment 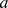, because making a high initial investment then yields high returns already from the first round of the interaction; consequently, individuals obtain higher payoffs by making high investments already from the beginning of the interaction. While the initial investment increases, the selection pressure for the conditional response decreases and finally reverses, as a strategy investing a large amount in the beginning and increasing investments even further in the following rounds may end up overinvesting. Eventually, after the reactivity 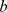 evolves close to 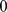 (falling below 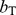), the initial investments 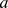 also evolve to 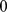. In this final phase, with very little reactivity, the dynamics simply resemble those of the continuous prisoner's dilemma, in which no cooperative investments can be maintained without additional mechanisms.

### Phases of the investment cycle

Next, we introduce a measure that helps us monitor the evolution of strategies along the investment cycle, and that suitably reduces the two-dimensional trait space, spanned by the two investment traits, to one dimension. For this purpose, we define cycle phases, 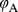 and 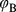 for Mutualist A and B, respectively, so that these monotonically increase along the investment cycle. As shown by the small arrows in Figure 1*B*, these phases are determined by the direction of the selection gradients (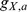,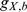) acting on the traits (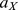, 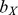) of Mutualist 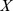 with 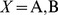.

Depending on the signs of 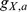 and 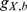, we can distinguish four quadrants of 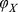, measured clockwise relative to the positive vertical axis. In the first quadrant, 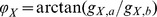; in the second quadrant, 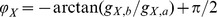; in the third quadrant, 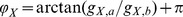; and in the fourth quadrant, 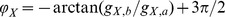. The boundaries between these phases thus correspond to evolutionary isoclines, i.e., to curves in the trait space along which the selection pressure vanishes for either one of the two traits.

Phase I is characterized by positive selection pressures on 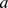 and 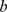, so that both trait values and investment levels increase (phase I in Figure 1*B* and 1*C*, 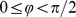). In phase II, while trait 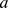 still increases, trait 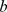 declines, as the selection pressure on 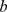 is negative (phase II in Figure 1*B* and 1*C*, 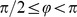). In phase III, more exploitative strategies, which invest less and thus gain more, are favored by selection, so that investment levels evolve to 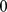, as traits 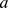 and 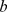 both decline (phase III in Figure 1*B and C*, 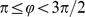). For low degrees of polymorphism, selection gradients in the fourth quadrant rarely occur; here, trait 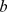 would grow while trait 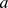 would shrink (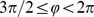).

Figure 1 shows that the cycle phase derived from the selection gradients acting on Mutualists A and B adequately indicates the direction of evolutionary dynamics along the investment cycle, in monomorphic populations or in populations with a low degree of polymorphism.

### Phase polymorphism

**Figure 2:**
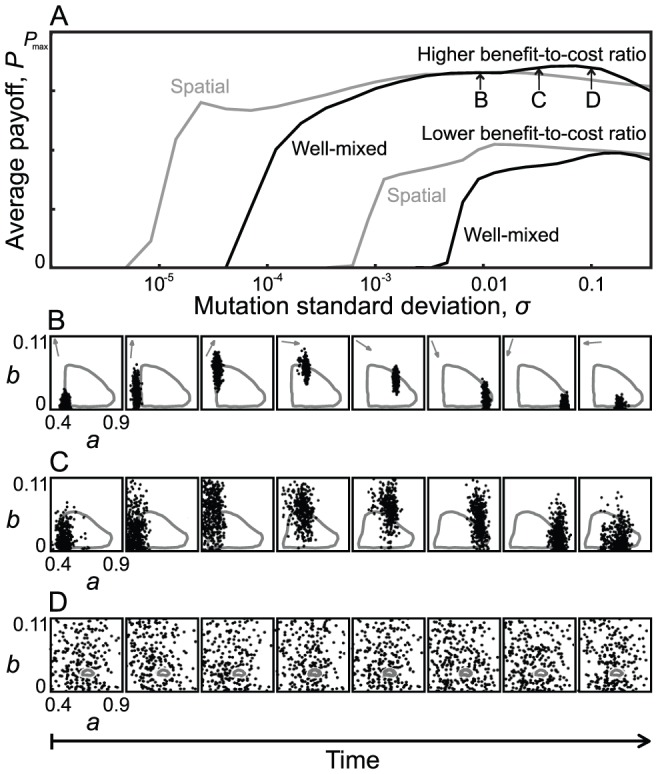
Evolution and stability of mutualistic investments in communities with higher degrees of polymorphism. (A) Diversity thresholds revealed by the effect of mutational variability on the average payoff in the community. For lower mutational standard deviations 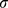, there is no mutualism (left-hand side), while stable community-level mutualism evolves abruptly once mutational variability is high enough (right-hand side). Results are averaged over the two mutualists and 15 replicate model runs. Payoffs can range between 0 and the maximal potential payoff 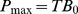. (B, C, D) Polymorphic spread of strategies in well-mixed communities, and their evolution along the investment cycle, with low, medium, or high mutational standard deviations: 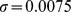 in B, 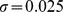 in C, and 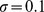 in D. As the averages of the traits 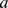 and 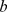 move along the investment cycle, they trace out the shown circular lines, corresponding to cyclic oscillations whose amplitudes decrease as 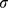 increases. Other parameters: 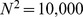 in A and 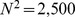 in B, C, and D; lower benefit-to-cost ratio of 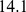 in A: 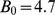, 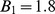, 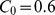; higher benefit-to-cost ratio of 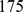 in A, B, C, and D: 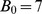, 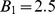, 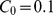; 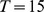, 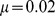.

In the next step of our analysis, we allow higher degrees of polymorphism. As shown in the previous section, when mutation probability and/or mutation variance are low, the polymorphic spread among strategies remains narrow, as the two mutualist communities evolve along the investment cycle (Figure 2*A*, left-hand side). However, there is a sharp transition in the outcome as the variety of mutants increases. Above a critical supply of strategy diversity, the two polymorphic populations can perpetually maintain strategies that on average are mutualistic and that lead to a high and stable level of average payoff (Figure 2*A*, right-hand side). This stable community-level mutualism still implies cyclic behavior, as the averages of both investment traits gradually evolve along the investment cycle also in populations with higher degrees of polymorphism (Figure 2*B*–*D*). Importantly, however, with the increase of mutational variability, this cyclic behavior becomes perpetual, as the evolutionary dynamics no longer collapse to zero investments at the end of phase III. The increase of mutational variance not only affects the polymorphic spread of strategies along the investment cycle, but also its shape and amplitude (observe the decrease of cycle amplitude with the increase of 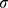 in Figure 2*B*–*D*). To understand these effects of mutational variability, we need to appreciate, first, how and why polymorphism arises, and second, what it implies for the community-level stability of mutualistic interactions. For this, it is helpful again to consider phases and selection gradients along the investment cycle.

**Figure 3:**
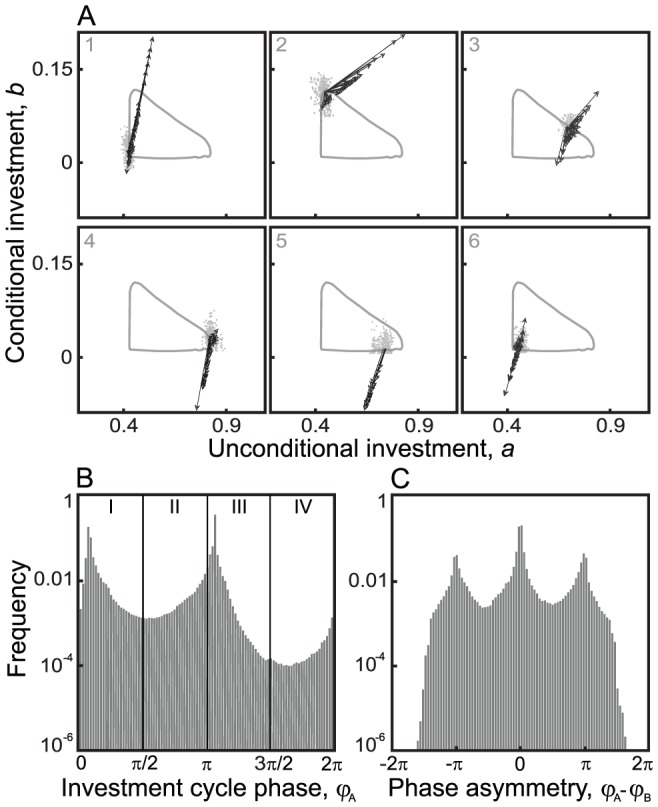
Selection gradients along the investment cycle and resultant phase distribution in a well-mixed polymorphic community. (A) Arrows indicate the selection gradients on a random subsample of individuals of Mutualist A as that mutualist's trait distribution (gray dots) moves along the investment cycle (gray circular lines). Average long-term polymorphic distribution of (B) phases along the investment cycle and (C) corresponding phase asymmetries during the evolution of mutualistic investments, averaged over three replicate model runs and shown on logarithmic scales. The phase asymmetry in pairs of interacting individuals of Mutualist A and B is measured as the difference of their phases, 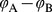. The peaks at 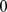 and 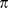 in B correspond, respectively, to the vertical and horizontal edges of the investment cycle. Parameters: 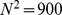 in A and 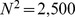 in B and C; 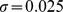 in A and 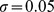 in B and C; 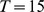, 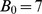, 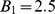, 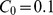, and 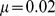.

Individuals in polymorphic populations encounter a diverse set of strategies, so the selection gradients they experience need to be determined accordingly: 
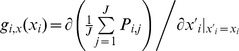
, where 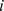 is the focal individual, the sum extends over all individuals 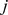 of the other mutualist, and the parenthesis encloses the expected payoff of a mutant offspring of individual 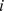 with strategy 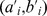. These selection gradients, shown as arrows in Figure 3*A*, help us understand the emergence of cyclic dynamics and phase polymorphism. At the beginning of the investment cycle (phase I), mutations will typically cause some symmetry breaking between the investment strategies of the two mutualists, while the polymorphic spread among strategies still remains narrow (Figure 3*A*, Panel 1). Once a trajectory reaches phase II, the selection pressures on the two 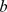 traits approach 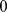, making them especially susceptible to neutral drift, and thus enhancing the symmetry breaking and polymorphic spread (observe the diversity of gradient angles in Figure 3*A*, Panel 2). Similar mechanisms operate at the boundary between phases II and III, where selection pressures become weak on the 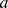 traits (Figure 3*A*, Panels 3 and 4). Finally, when a trajectory reaches phase III (Figure 3*A*, Panel 5), the strongest effect occurs: when traits evolve close to the boundary that separates trait combinations corresponding to phases III and I (see the partially overlapping black and thick gray lines in Figure 1*B*), mutations can take the two traits across the boundary, from phase III to I and back. Such a jump across the boundary changes the sign of the selection gradient for both of the traits for at least one of the mutualists (Figure 3*A*, Panels 6 and 1). This causes recurrent transitions across the boundary, so trajectories linger at this boundary, which naturally increases their polymorphic spread. Once a sufficient proportion of the population has thus traversed the boundary, the investment cycle is retriggered (Figure 3*A*, Panel 1). Notice that the degree of phase polymorphism varies along the investment cycle. It typically decreases in the middle of phases I and III (observe how all gradients are pointing in essentially just one direction in Figure 3*A*, Panels 1 and 5), and increases at the boundaries between the phases (observe the diversity of gradient angles in Figure 3*A*, Panels 2, 3, 4, 6).

With further increases of mutational variability, even higher levels of polymorphism develop, so strategies diffuse across all phases of the investment cycle. In highly polymorphic populations, as a consequence of this phase spread, selection pressures become widely different for different parts of the populations; hence, a wide variety of strategies becomes established, ranging all the way from phase I to phase IV (Figure 3*B*). Competition between strategies and strategy pairs shapes the phase distribution of the community (Figure 3*B* and *C*), as individuals or pairs with a competitive disadvantage fade out from the community. These losing strategies are typically those at the beginning of phase I or at the end of phase III (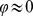 or 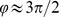), as well as strategy pairs with an extreme asymmetry or exploitation (at the tails of the distribution in Figure 3*C*). The two most successful, and hence most frequent strategies, are conditional cooperators (akin to Tit-for-Tat strategies, with high 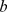 and low 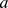; Figure 3*B*, peak close to 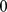) and unconditional cooperators (akin to All-C strategies, with high 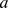 and low 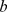; Figure 3*B*, peak close to 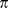). The result of competition within the polymorphic populations is thus a diverse cast of interactions, ranging from strongly mutualistic (central peak in Figure 3*C*, corresponding to both mutualists being in the same phase) to exploitative (two lateral peaks in Figure 3*C*, corresponding to one mutualist being in phase I and the other in phase III, or vice versa).

We highlight that the results depicted in Figure 2*A* are essentially invariant for lower mutation rates (not shown). The intuitive explanation is that such lower mutation rates have two effects. First, there are fewer mutations occurring in any given time window, which by itself would hinder the retriggering of the investment cycle. Second, the pace of directional evolution slows down for such lower rates, so the trait distribution lingers for longer periods at the phase boundaries, which by itself would facilitate the retriggering of the investment cycle. These two effects essentially cancel, leaving the critical levels of mutational variability needed for retriggering the investment cycle largely independent of the considered mutation rates. By contrast, this retriggering is strongly affected by the benefit-to-cost ratio. When the benefit-to-cost ratio is large, a smaller amount of mutational variability suffices to maintain strategy polymorphism and thus community-level mutualism (Figure 2*A*, compare upper and lower pairs of curves). Moreover, localized interactions and limited dispersal promote strategy polymorphism, by creating a spatial mosaic structure, as we will describe in more detail in the next section. Accordingly, in spatially structured populations the transition to stable community-level mutualism appears at lower mutational variability (Figure 2*A*, compare gray to black pairs of curves).

### Spatial bubbles and polymorphism

**Figure 4:**
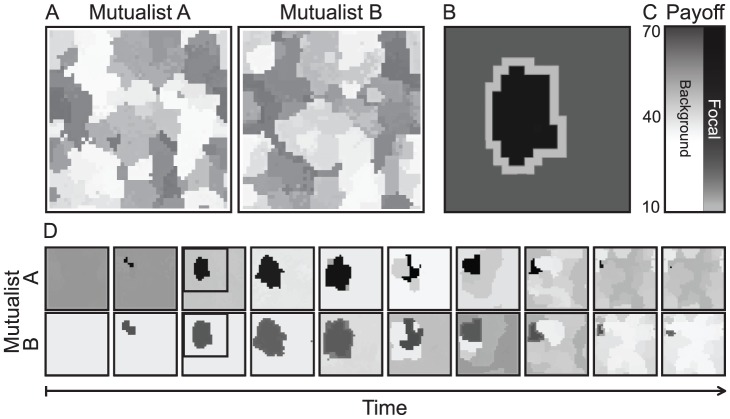
Spatial bubble dynamics and appearance of the insulating boundary layer. (A) Typical snapshot of the spatial mosaic structure, indicating a high degree of polymorphism and spatial bubbles comprising different strategies. Each pixel represents an individual, rendered according to its payoff between zero (light gray) and the maximal value (black). (B) Enlargement of a bubble with its surrounding insulating boundary layer. Notice that individuals inside and outside the bubble both have higher payoffs than the individuals forming the boundary layer. This panel is obtained as an overlay of Mutualist A and B from the third column in D according to their average payoff values. (C) Shading of background strategies ranges from white to mid-gray, while shading for the focal bubble ranges from dark-gray to black, as the payoffs of individuals increase. (D) Time series of snapshots for a spatial bubble (black to dark-gray shading) that first expands and then vanishes, illustrating a spatial “boom and bust” cycle (snapshots are taken in generations 3013, 3040, 3260, 3399, 3493, 3625, 5165, 5620, 6400, and 6408). Parameters: 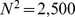 in A and 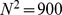 in D; 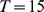, 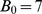, 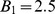, 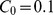, 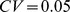, and 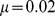.

In spatially structured mutualistic communities with local interactions and limited dispersal, strategy polymorphism occurs together with a dynamic spatial mosaic structure (Figure 4*A*) of spatially abutting “bubbles.” Here we use the term “bubble” to describe spatial clusters that are compact and contiguous, contain similar strategies on the inside and different ones on the outside (Figure 4*B*), and grow gradually in size from a small core before disappearing through a sudden collapse (Figure 4*D*). For the most part, there is a strong correspondence between Mutualist A and Mutualist B with regard to the position and extent of spatial bubbles, and typically the corresponding strategies are asymmetric, giving one species a higher payoff than the other (compare the shading of corresponding sites in Figure 4*A*). To fully understand the role of spatial population structure in stabilizing mutualism, we thus have to understand the composition of, and the ongoing dynamics among and within, these bubbles.

**Figure 5:**
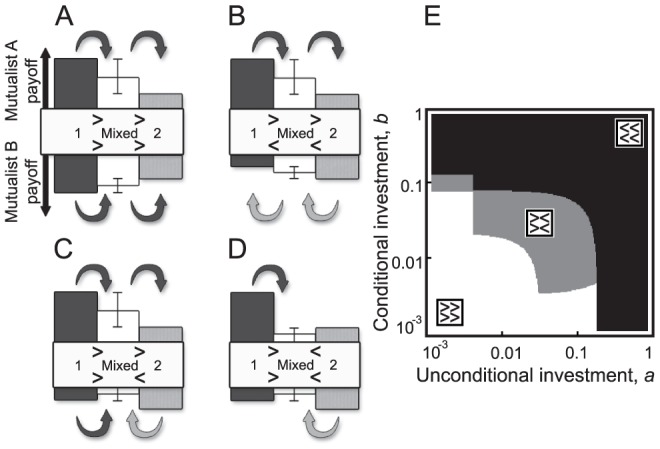
Possible replacement dynamics at the interface between two spatial bubbles. We presume that strategy pairs meet at the interface (white columns) of bubble 1 (dark-gray columns) and bubble 2 (light-gray columns). Here, the following cases can occur: (A) Unidirectional invasion: both mutualists from bubble 1 invade the other bubble, as both A1 and B1 have a higher payoff than A2 and B2. (B) Partner swapping: A1 has a higher payoff and outcompetes A2, but B2 has a higher payoff and outcompetes B1, hence A1 pairs up with B2. (C) Catalyzed invasion: only A1 is able to outcompete its competitor from bubble 2, but as it spreads, it makes it possible for B1 to follow. This is feasible because B2 fares worse with A1 than with A2, so as A1 spreads, the payoff of B2 decreases with its new partner, and hence B1 can now invade. (D) Insulating boundary layer: at the interface of two spatial bubbles, the originally competitively superior strategies A1 and B2 enter the interface, but as both then have a lower payoff than with their original partners, neither can spread further. Column heights depict the payoffs of strategies. For the described dynamics, the payoffs of a strategy with its two possible partners (i.e., from either bubble 1 or 2) at the interface must lie within the interval indicated by the two whiskers in the middle column. (E) Invasion dynamics depend on the strategy compositions of the mutualist pairs. Formation of an insulating boundary layer is the result of the encounter of two strategy pairs (A1&B1, A2&B2) that are mutually unable to invade each other (gray area). Otherwise, one bubble invades and replaces the other (in the white area, the strategy pair of bubble 1 wins, whereas in the black area, the strategy pair of bubble 2 wins). We evaluate these outcomes in the absence of evolution (no mutations) and for one strategy pair (A1&B1) initially occupying one half of the lattice and the other strategy pair (A2&B2) occupying the other half. Parameters: A1 and B1, 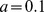 and 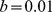; A2, 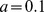 and 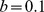; B2, 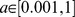 and 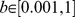; 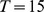, 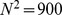, 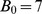, 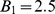, and 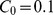.

As we saw in the previous section, symmetry breaking and phase polymorphism along the investment cycle can lead to asymmetry between the mutualistic partners. This emerging asymmetry is strongly exaggerated by the spatial bubble structure, as competitively inferior strategies vanish quickly, while exploiting strategies are likely to attempt an invasion of adjacent bubbles, supported by their high payoffs. Hence, spatial bubbles are often composed of exploiting strategies and their exploited partners. The degree of asymmetry and its trend among bubbles can vary, and this diversity of asymmetries provides the stage for bubbles expanding, splitting, or collapsing in various ways (Figure 5). If a strategy can outcompete that of a neighboring strategy, its successful invasion further depends on its maintaining its competitive superiority in the invaded patch. Hence, invasion success can be determined by considering the relative payoff of the invader before and after invasion.

To demonstrate this, we consider the interface between two bubbles as the site where strategy pairs can meet. We can then analyze all possible dynamics at this interface. We label the two bubbles so that Mutualist A has a higher payoff (>) in bubble 1 than in bubble 2. We can neglect cases with equal payoffs in the two bubbles, as these do not change the configuration of strategies, and thus do not contribute to the bubble dynamics. Relations between the payoffs in bubble 1, at the interface, and in bubble 2 (Figure 5) can thus be represented as 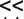, 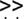, 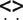, or 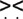 for Mutualist A, and by 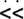, 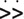, 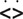, or 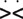 for Mutualist B, yielding seven distinct situations: 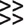, 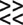, 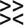, 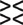, 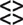, 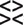, 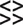. Corresponding to Figure 5, the upper row in these stacked symbols refers to Mutualist A and the lower row to Mutualist B, while the first column refers to the payoff comparison between bubble 1 and the interface, and the second column to the interface and bubble 2. The first four cases, in which Mutualist A in bubble 1 always has a higher fitness than Mutualist A at the interface (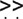), correspond to replacement dynamics (Figure 5*A*–*D*) involving unidirectional invasion (Figure 5*A*), partner swapping (Figure 5*B*), catalyzed invasion (Figure 5*C*), and coexistence of the two bubbles (Figure 5*D*). In the last three cases, Mutualist A has a higher payoff at the interface than in either bubble (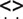). We can interpret these situations as having a bubble with a strategy pair formed at the interface that can spread in both directions. The resultant new pairs of adjacent bubbles will then behave in one of the ways covered by the first four cases above. Thus, the four cases shown in Figure 5*A–D* and discussed in more detail in that figure's caption cover all possible dynamics between the two bubbles.

**Figure 6:**
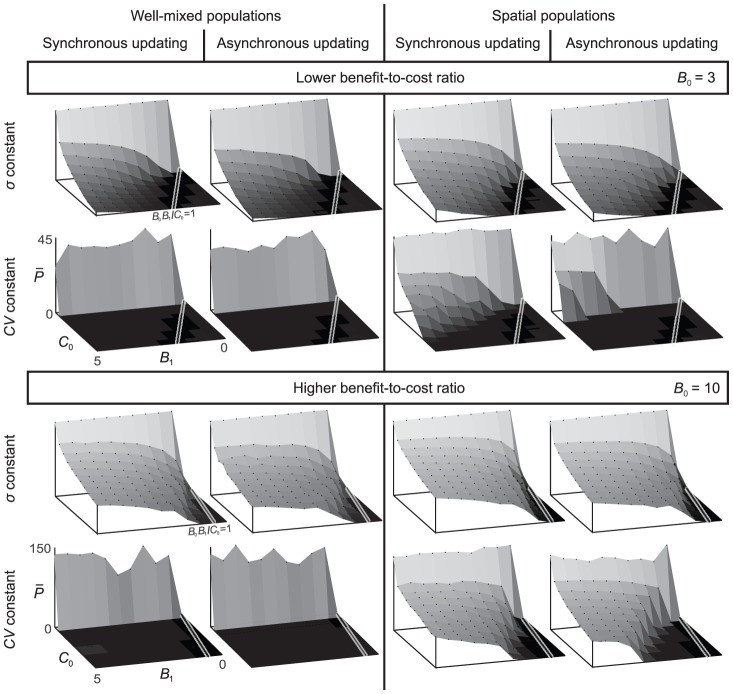
Average payoff as a function of the parameters of the cost and benefit functions, spatial structure, and update rules. Each individual panel shows the average payoff of Mutualist A and Mutualist B, calculated as the arithmetic mean of their payoffs over the last 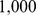 generations, out of the total of 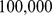 generations, and averaged over five replicate model runs. The three parameters of the benefit and cost functions are varied as follows: 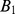 and 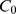 along the axes and 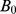 between the upper (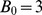) and lower (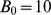) eight panels. The black line on white background indicates the 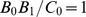 threshold, below which no investments can evolve. Results for well-mixed populations are shown in the eight panels on the left, while results for spatially structured populations are shown in the eight panels on the right. Odd and even columns correspond to synchronous and asynchronous updating, respectively. Rows show results for a constant mutational standard deviation 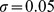 (first and third rows) and a constant mutational coefficient of variation 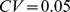 (second and fourth rows). Other parameters: 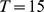, 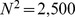, and 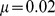.

**Figure 7:**
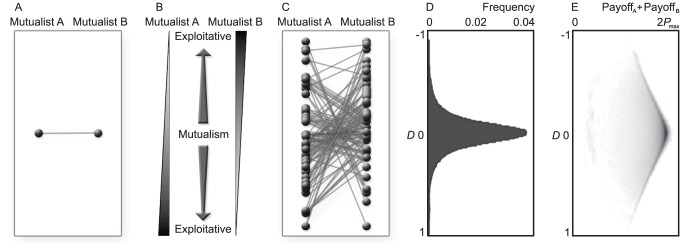
Schematic representation of mutualistic interactions in (A) monomorphic and (C) polymorphic mutualist communities. Spheres depict strategies, and the links between spheres represent the interactions between interacting strategies from the two mutualist guilds. (B) According to its own and its partner's strategy, an individual receives a payoff (schematically illustrated by two triangles that become darker and wider as the received payoff increases). The comparison of payoffs between partners shows whether their interaction is more mutualistic (middle) or more exploitative (bottom and top). (D) Average distribution of interaction types in our model, showing that small relative differences 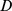 between the payoffs of interacting individuals are more common or longer-lasting than extreme exploitations. (E) Average distribution of the payoff sums and relative payoff differences for interacting individuals of Mutualist A and Mutualist B, demonstrating that, on average, payoffs in asymmetric, or exploitative, interactions are lower than in symmetric, or more mutualistic, interactions. The distributions in D and E are based on sampling all individuals in every 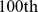 generation for 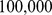 generations and for five replicate model runs. The relative difference between the payoffs 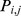 and 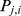 of individuals 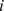 and 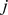 is given by 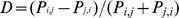, and 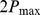 is given by 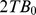. Parameters: 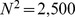, 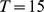, 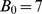, 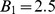, 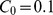, 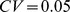, and 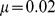.

The most relevant case for preserving phase polymorphism occurs when the two exploiting strategies of two adjacent bubbles, having high payoffs within their bubble, can both enter the intervening interface, but their exploited partners cannot (Figure 5*D*). Then, these exploiting strategies meet at the interface, but are mismatched: by interacting with each other, they experience lower payoffs compared to when they interact with their original partners. Consequently, neither bubble can invade the other (under deterministic updating), and an insulating boundary layer forms between them (Figure 5*D*). These effects yield a relatively static mosaic structure, in which most bubbles are separated by insulating boundary layers, which in turn fosters the long-term coexistence of a diverse set of strategies in both mutualist guilds. Nevertheless, the resulting mosaics are eventually not immune to the degradation of mutualism within bubbles, as strategy pairs evolve along the investment cycle, making the mosaic structure (if only slowly) dynamic.

The dynamics of the spatial mosaic are governed by evolutionary processes that maintain a balance between the expansion or emergence and the contraction or collapse of bubbles. First of all, inside a bubble, evolution drives strategies through the investment cycle. Sooner or later, this stochastic evolution changes the strategy pairs of two neighboring bubbles in such a way that their boundary layer (Figure 5*E*, gray area) loses its insulating property, thus enabling invasion from one bubble to the other (Figure 5*E*, white or black areas). Although this invasion itself is a rapid process, the evolutionary time that is required for the insulating boundary layer to break down is usually long. Counteracting mechanisms can restore the loss of diversity resulting from bubble collapse: this happens through the emergence of new bubbles as a result of successfully established mutations (if such a mutant conquers only part of a bubble) or through the fragmentation of existing bubbles. In the latter case, mutants occurring within the insulating boundary layer are able to invade either one of the adjacent bubbles. Through this invasion, the mutant opens up the boundary and can catalyze the invasion of strategies from the neighboring bubble (similar to how Mutualist A1 catalyzes the invasion of B1, as in Figure 5*C*). Thus, while the two neighboring bubbles could originally not invade each other, this becomes possible through the mutant serving as a “third party.” The resultant expansion of the invading bubble can then split the invaded bubble (Figure 4*D*, from fifth the column onwards), upon which the two resultant parts can take separate evolutionary paths.

In summary, strategy diversity, and thus, community-level mutualism, is efficiently stabilized through the formation of an insulating boundary layer between bubbles of strategies. This would result in a static mosaic structure, which, however, becomes dynamic as strategies evolve along the investment cycle. The invasions resulting from these stochastic evolutionary processes establish a balance between the emergence and collapse of bubbles that maintains a level of polymorphism in a more efficient way than the corresponding well-mixed mutualistic community. The diversity threshold for community-level mutualism is thus more easily passed in spatially structured communities (Figure 2*A*).

### Extended analysis of the Doebeli-Knowlton model

In the light of our understanding of the evolution and stability of interspecific cooperative investments established in the previous sections, we can now revisit, complement, and extend the pioneering investigation of Doebeli and Knowlton (DK) [43].

Specifically, we can present a more comprehensive and systematic overview of the evolution of interspecific cooperative investments under various relevant conditions (Figure 6). First, we present the necessary condition that no mutualistic investments can evolve below 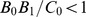, that is, when the benefit-to-cost ratio falls below 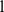 and mutualism is thus not advantageous (see thin black lines with white background in Figure 6), which in general holds under all conditions. Also, the transition to high stable levels of mutualistic investments is sharper for higher benefit-to-cost ratio (rows with 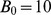 in Figure 6). Second, besides the synchronous updating that was originally applied by DK in the spatial model, we also consider asynchronous updating, and find that mutualism is unstable for a wider range of benefit-to-cost ratios (compare third and fourth columns in Figure 6). Third, we test different competition rules under asynchronous updating, such as the pairwise comparison rule instead of the best-takes-over rule used by DK, and we can conclude that outcomes are essentially unaffected by these different rules (not shown). Fourth, we consider two methods for generating mutant traits. In one version, as in the original DK model, we draw the trait values of mutants with a constant coefficient of variation, so that the mutational standard deviation linearly increases with the current trait value (row labeled “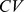 constant” in Figure 6). With this approach, mutational variance for small trait values becomes very low, equaling 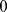 when trait values equal 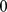. Here we examine a different assumption, according to which mutational variance is constant for all trait values (rows labeled “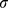 constant” in Figure 6). Comparing the results, we arrive at an important conclusion, namely, that the qualitative contrast reported by DK between “gradual evolutionary decay of cooperation” in the well-mixed model and “long term persistence of mutualism” in the spatially structured model is restricted to the assumption that mutations have a constant coefficient of variation (“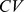 constant” in Figure 6). Notably, our investigation reveals that relaxing this assumption, by assuming constant and medium levels of mutational variance, mutualism robustly evolves for all kinds of populations structures and update rules (compare “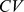 constant” vs. “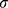 constant” in Figure 6). We note here that our results are qualitatively robust to changes of the number 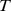 of iterations during the mutualistic interaction, which we have confirmed by examining shorter (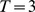) and longer (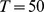) interactions instead of 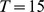 (not shown). Fifth, we demonstrate that below a threshold level of mutational variability no stable levels of mutualistic investments evolve in the community, and this threshold is considerably lower for higher benefit-to-cost ratios and for spatially structured populations (Figure 2*A*). In summary, we conclude that spatial population structure has a beneficial effect on the evolution of stable high interspecific investment levels, but this effect is only apparent for constant 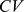, for small mutational variability, and for small benefit-to-cost ratios. By changing these conditions, mutualism can be stable both in well-mixed and in spatially structured communities.

Finally, our results enable us to understand the mechanisms underlying the evolution and stability of mutualism in greater depth. In particular, we can highlight several new mechanisms for stabilizing mutualism, both in well-mixed and spatially structured populations. First and foremost, we have presented the investment cycle (Figure 1), which drives the main coevolutionary dynamics of traits, and underlies the evolution of cooperative investments levels in mutualist communities. While the cyclic dynamics can already be seen in DK's results (e.g., in their Figure 2), here we have put it into the spotlight of our analysis. Second, we have demonstrated the spreading of the investment cycle phases, and have revealed the diverse ways strategies interact when they are in different phases, both within and between mutualist guilds (see Figure 2 and Figure 5). Thus, in contrast to the interpretation of DK, that mutualism is maintained by a balance between the “continual reoccurrence of mutualistic types” and then a “gradual evolutionary decay of cooperation” (DK), we show that mutualism is mainly stabilized by phase polymorphism along the investment cycle (Figures 2, 3, and 4). The emerging phase polymorphism and underlying strategy diversity recurrently retrigger evolutionarily increasing levels of cooperative investments in some portion of the community (phase I in Figure 3*A, B*), a process that is essential for maintaining high investment levels. Third, while DK already noticed “considerable genetic heterogeneity,” here we have demonstrated the existence of sharp diversity thresholds. In addition, we can provide an explanation for the differences in the stability of mutualism under constant 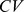 vs. constant 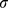, as well as under low 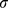 vs. high 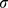. These differences derive from the fact that if phase polymorphism is largely lost, it is much harder to retrigger the investment cycle (by attaining trait combinations above 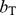) once the community has reached the last phase of the investment cycle (or in other words, once phase I has vanished from the community). For similar reasons, any mechanism that prevents or counteracts the generation of phase polymorphism will increase the chances of losing mutualism. Whereas DK suggested that “for mutualism to evolve,… spatial structure… is required,” here we have reversed that logic, by clarifying that strategy diversity and phase polymorphism along the investment cycle are responsible for maintaining high investment levels and that the only effect of spatial structure is to enhance this polymorphism. Fourth, we have studied spatial mosaic dynamics by analyzing replacement dynamics in the mutualist populations to understand why spatial structure increases polymorphism (Figure 5). Combining our insights with DK's intuitive concept of the “boiling sea of mutualistic bubbles,” our investigation reveals the complex dynamics among bubbles and the key role of the insulating boundary layer in preserving polymorphism in spatially structured populations. Fifth, this enables us to understand why asynchronous updating makes mutualism less stable, as it more easily shatters insulating boundaries, promotes asymmetric and uncoupled invasion of the two mutualists among bubbles, and hence makes the homogenization of bubbles more likely. Sixth, while our results explain why spatial structure is helpful in maintaining mutualism, they also demonstrate that space itself does not always suffice, and neither always is necessary, to maintain community-level mutualism.

## Discussion

Here we have revealed several fundamentally new mechanisms for the maintenance of interspecific cooperation. We show that pairs of strategies evolve through investment cycles, which on their own always result in full defection. Our analyses demonstrate, however, that in both well-mixed and spatially structured communities mutualisms can be perpetually stable if a strategy-diversity threshold is exceeded and sufficient polymorphism is generated and maintained in the community. In other words, such a polymorphism of investment strategies is the main factor stabilizing mutualism. Compared to the well-mixed case, a lower amount of variation suffices to maintain mutualism in spatially structured populations; we have shown that this is because of insulating boundary layers that promote polymorphism by preserving spatial bubbles of matched mutualistic strategies. Our findings underscore that mutualism is not always a stationary outcome, but may involve a polymorphism of investment levels that vary both in space and in time (Figure 7).

### Importance of polymorphism

Our analysis has shown that when our model community exhibits a stable mean level of mutualism, it is invariably characterized by a high degree of polymorphism, and that mutualism persists only if this polymorphism is maintained. Without strategy polymorphism, the evolutionarily stable state of the system is a community consisting only of full defectors (no investment). This is because full defection is the best response to itself, and no mutant investing more can spread in either species [53]. No other strategy pairs are best responses to each other, so there are no other evolutionarily stable states. However, there are many pairs of strategies that can spread in initially non-mutualistic populations (Figure 1); above a threshold of reciprocating investments, evolution guides these strategies through an investment cycle, which eventually always results in no investment. Hence, mutualistic investments in our model are fundamentally unstable [56], never reaching finite stable levels even though they may initially be increasing. This means that in our model evolution without strategy polymorphism can only temporarily lead to high mutualistic investments before these eventually collapse again.

Similar dynamics have been observed in studies investigating the evolution of intraspecific cooperative investments in different game-theoretical models. For example, in the prisoner's dilemma game with discrete reactive strategies [4], [57], the Tit-for-Tat strategy (TFT) can oust the always-defect strategy (All-D), but the always-cooperate strategy (All-C) can spread in a population adopting TFT, which in turn enables invasion by All-D. As mentioned in the Results section, TFT is similar to strategies with high conditional investments 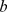 in our model, whereas All-C is similar to strategies with dominating unconditional investments 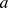. Without the continuous reestablishment of strategies by mutations, models with discrete strategies may also end up in a fully defective state [4], [57].

In contrast to these results for communities with low degrees of polymorphism, when sufficient polymorphism is generated, community-level mutualism becomes stable. For this to happen, the degree of polymorphism needs to exceed a threshold (Figure 2*A*). Even in well-mixed populations, stochastic symmetry breaking in the interactions, combined with phase polymorphism along the investment cycle, leads to the emergence of a high variety of strategy pairings and payoffs (Figure 3*B, C*). While evolution drives individual strategy pairs toward exploitation (and, ultimately, to zero investment), the exploited partner has a fitness disadvantage: consequently, the highly exploitative pairs are replaced by more mutualistic pairs, which show less asymmetry in their payoffs (Figure 3*C*). These pairs are typically composed of strategies from phase I of the investment cycle (Figure 3). Our findings thus indicate that the interspecific interactions exist in a state of permanent flux, fluctuating between different investment levels at the individual level. In contrast, the mean level of mutualistic investment remains positive (and for high degrees of polymorphism becomes stable), shaped by a balance between two components of selection: strategy evolution along the investment cycle and replacement of overly exploited strategies and of mismatched strategy pairs.

Spatial population structure further facilitates the stability of mutualism by playing a key role in supporting polymorphism (Figure 4 and 5). However, limited dispersal and localized interaction alone do not maintain mutualism, but only when they work together with mutational variance that is high enough to sustain a critical level of polymorphism (Figure 2*A*). In spatially structured populations, the interaction among emerging, invading, and collapsing spatial bubbles of strategy pairs creates a dynamic spatial mosaic, by means of which different phases of the investment cycle are distributed among bubbles. As a result of this phase spread, the evolutionary dynamics of mutualistic investments become decoherent among the different bubbles. This is called phase diffusion, which in general occurs when stochastic drift reduces correlations among the cycle phases of subsystems (here the spatial bubbles) comprising a system (here the full community). Consequently, among bubbles, the community shows a wide but stable range of interaction types along the mutualism–exploitation continuum (Figure 7*D*). We have shown how mechanisms operating at the interface of these bubbles effectively prevent the spatial homogenization of strategies across the community by creating insulating boundary layers (Figure 4*B* and 5*D*) that in turn sustain the spatial mosaic structure of bubbles together with the implied strategy polymorphism. We emphasize here that the mechanism of spatial population dynamics and interaction between neighboring bubbles described here fundamentally differs from previously described roles of spatial structure in models of intraspecific cooperation (in which, in a nutshell, cooperation is maintained by the clustering of cooperators and by their spatial segregation from defectors [49]).

### Complexity of mutualisms in nature

One implication of our study is that the diversity of mutualistic strategies in natural communities may be high not only because of mutation and recombination, or inherent species diversity, but also as a product of selection pressures resulting from the complex dynamics of mutualistic interactions occurring within polymorphic mutualist guilds. We note here that, based on our model assumptions, the two mutualist populations may correspond not only to single species interacting pairwise, but also to two interacting mutualist guilds [58], that is, a collection of species with the same function in mutually beneficial ecological interactions. Thus, strategy polymorphism in our model can relate not only to variation within, but also across, species. Indeed, growing empirical evidence suggests that polymorphisms of mutualistic investment strategies are common in nature [19], [21], [22], [27], [34], [58]–[66], even on small spatial scales [67], [68]. Many studies suggest that microbial populations and communities are often structurally and genetically more diverse [67], [69], considering both type or strain richness and/or genetic diversity [68], than what can be explained by local host diversity [70]. Also the effectiveness of rhizobia, such as their ability to form nodules and their capacity to fix nitrogen, varies greatly within species, and naturally, between species [27], [58], [71], [72]; similar conclusions hold for the performance of mycorrhizal interactions [26], [65], [73]. This diversity amounts to a high variety of investment strategies; in other words, less mutualistic types coexist with more beneficial mutualists in natural communities [6], [19], [21], [22], [27], [34], [36], [71].

Mutualistic interactions are known to shift along the mutualism–exploitation continuum in response to changes in environmental factors [7], [19], [58], [59], [74], [75]. For example, many nutritional mutualisms, including mycorrhizal or rhizobial mutualisms [13], are highly beneficial for host plants as long as the resource provided (e.g., phosphorus, nitrogen, or copper) is absent from the environment, but can become harmful (implying that costs exceed benefits) when that resource no longer is a limiting factor [2], [38], [19], [76]. This not only underscores the importance of reactive strategies for modeling mutualism, but also offers one explanation for the spatial mosaic structures observed that involve different genotypes, as well as the different local coevolutionary states shaped by different local selective forces [45], [65], [77]–[79]. Our findings highlight that spatial environmental heterogeneity is not required for the creation of such mosaics, as the mechanisms unraveled here provide a testable alternative explanation of these empirical observations, even in the complete absence of spatial environmental heterogeneity.

Mutualisms can also be unstable on a much longer time scale, and there can be a diversity of mutualistic, parasitic, and free-living variants within higher taxa. The phylogenetic analysis of mycorrhizal and free-living homobasidiomycetes suggests that there have been several transformations between symbiotic and free-living forms [80]. The gain and loss of mutualistic traits thus seems to be relatively common on an evolutionary time scale, a finding that is in good agreement with our model-based results.

### Limitations

The model by Doebeli and Knowlton [43] has been criticized for being applicable only to organisms with high cognitive abilities [13]. Yet it has been demonstrated that even the simplest unicellular organisms are capable of complex reactive behavior. For example, it has been shown that, in response to the concentration of received nutrients and synthesized products, hosts and symbionts can control their exchange of material simply by controlling fluxes through their various metabolic pathways [31], regulating and operating proteins [29], [81], or inducing structural changes at the host–symbiont interface [28], [82]. Such adjustments closely resemble the reactive, conditional nature of interspecific cooperative investments, as captured by the model we have analyzed here.

However, there are assumptions in our model that can and should be relaxed and modified in subsequent studies. For example, in the model studied here, one individual always interacts with only one partner. Yet, in the majority of examples in nature, one host can interact with several symbionts at the same time, and vice versa [12], [13], [27]. The square grid we have considered here might be suitable if both mutualists have limited dispersal and are thus spatially confined. Of course, one or both partners can be more motile, without well-mixed populations being the immediate result. Moreover, different interaction topologies could be considered, such as small-world or scale-free networks. Finally, partners in the current model have similar life cycles, which might apply only to a very limited number of biological examples; thus, assuming life-cycle asymmetries could be an important extension of the current model [43].

### Outlook

Our study has shown that the community-level picture of mutualism can be quite different from that at the individual level. As the mean outcome can provide misleading or poor information, a full understanding of the involved ecological and evolutionary dynamics requires an appreciation of the distribution of outcomes [40]. In line with various recent studies, we have demonstrated that mutually beneficial interspecific interactions should not be conceived only as (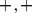) interactions, but as a continuous range of symmetrically beneficial (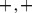), asymmetrically beneficial (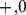), and explicitly exploitative or parasitic (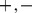) interactions [36] (Figures 3 and 7). Our results thus suggest that it is not enough to monitor average fitness advantages, as localized individual interactions may be situated at different points along the mutualism–parasitism continuum (Figure 7), and may also shift in time. The long-standing notion of mutualistic interactions being static is thus becoming extended as new findings, both experimental and theoretical, broaden our understanding. Consequently, exploitation and mutualism are not always strictly separate types of interactions, but in many instances may serve as boundaries of a continuous distribution of interactions between two mutualist guilds. This distribution reflects not only population or guild-level variation, but also dynamical changes of interactions occurring on ecological and evolutionary time scales.
